# INO80 participates in the pathogenesis of recurrent miscarriage by epigenetically regulating trophoblast migration and invasion

**DOI:** 10.1111/jcmm.16322

**Published:** 2021-03-16

**Authors:** Shu Xian, Yan Zhang, Li Wang, Fang Yao, Jinli Ding, Yanqing Wang, Xiao Yang, Fangfang Dai, Tailang Yin, Yanxiang Cheng

**Affiliations:** ^1^ Department of Gynecology and Obstetrics Renmin Hospital of Wuhan University Wuhan China; ^2^ Department of Obstetrics and Gynecology Ultrasound Renmin Hospital of Wuhan University Wuhan China; ^3^ Department of Clinical Laboratory Renmin Hospital of Wuhan University Wuhan China; ^4^ State Key Laboratory of Cardiovascular Disease Fuwai Hospital National Center for Cardiovascular Diseases Chinese Academy of Medical Sciences and Peking Union Medical College Beijing China; ^5^ Reproductive Medical Center Renmin Hospital of Wuhan University Wuhan China; ^6^ Department of Obstetrics and Gynecology Peking University People’s Hospital Beijing China

**Keywords:** INO80, invasive, recurrent miscarriage, trophoblast

## Abstract

The INO80 complex, a SWI/SNF family chromatin remodeler, has regulatory effects on ESC self‐renewal, somatic cell reprogramming and blastocyst development. However, the role of INO80 in regulating trophoblast cells and recurrent miscarriage (RM) remains elusive. To investigate the in vivo effects of Ino80 in embryo development, we disrupted Ino80 in C57 mice, which resulted in embryonic lethality. Silencing of Ino80 led to decreased survival capacity, migration and invasion of trophoblasts. Furthermore, RNA high‐throughput sequencing (RNA‐seq) revealed that Ino80 silencing closely resembled the gene expression changes in RM tissues. To investigate the mechanisms for these results, RNA‐seq combined with high‐throughput sequencing (ChIP‐seq) was used in trophoblast cells, and it showed that Ino80 physically occupies promoter regions to affect the expression of invasion‐associated genes. Last, Western blotting analyses and immunofluorescence staining revealed that the content of INO80 was reduced in RM patients compared to in healthy controls. This study indicates that INO80 has a specific regulatory effect on the viability, migration and invasion of trophoblast cells. Combined with its regulation of the expression of invasion‐associated genes, it has been proposed that epigenetic regulation plays an important role in the occurrence of RM, potentially informing RM therapeutic strategies.

## 1 INTRODUCTION

The processes of blastocyst implantation and placenta formation, which rely on the invasion of trophoblast cells, are the keys to initiating and maintaining human pregnancy. The placental trophoblast cells constantly invade into the maternal endometrium, where they anchor the embryo and form the placenta.^1^ The placenta, a place for oxygen exchange, nutrient uptake and waste removal, is essential for foetal development in the uterus. Failure of placental trophoblast cell invasion or arterial remodelling in the developing decidua, may result in hypertension and miscarriage, and these processes are controlled by complex epigenetic modification networks.^2^, ^3^

Recurrent miscarriage (RM), defined by two or more failed clinical pregnancies before 20 weeks of gestation, is a distressing clinical problem accompanied by social and economic impacts. It occurs in approximately 5% of couples attempting to bear children.^4^, ^5^ Frequently, the cause is multifactorial or elusive, which makes the couple and doctors feel frustrated. Several hypotheses for the aetiology have been proposed, such as chromosomal and uterine anatomic abnormalities, cervical insufficiency, alloimmune causes, endocrine abnormalities, antiphospholipid syndrome and exposure to environmental factors. Unfortunately, up to 50% of patients with RM do not have a clearly defined aetiology and are unable to have a successful subsequent pregnancy due to the lack of effective treatment strategies.^6^, ^7^

Extravillous trophoblast cells (EVTs) are differentiated trophoblast cells that migrate from the distal region of the placental villi column and invade into the interstitial space of the maternal uterine wall to successfully establish a human pregnancy.^8^ Impaired EVT migration and invasion are commonly associated with multiple pregnancy‐related diseases.^9^, ^10^ Therefore, a better understanding of the epigenetic regulation mechanisms of trophoblast invasion and identification of the key signalling networks behind this process are necessary for clinical diagnosis and treatment.

ATP‐dependent chromatin remodelling complexes are classified into four families, based upon the sequence homology of their ATPase subunit: SWI/SNF, ISWI, CHD and INO80.^11^ INO80, a member of the INO80 subfamily of chromatin remodelling complexes, is highly conserved from Saccharomyces cerevisiae to humans. Similar to other chromatin remodelling complexes, INO80 possesses ATPase and nucleosome sliding activity. Thus, INO80 regulates a variety of nuclear processes by depositing, moving, evicting or selectively altering nucleosomes in an ATP‐dependent manner.^12^ In turn, it has been implicated in many crucial biological functions, such as transcription regulation, DNA damage repair, DNA replication, chromosome segregation and telomere maintenance.^13^, ^14^, ^15^, ^16^

Although much has been done to characterize the functions of INO80, its specific role in embryonic development remains undefined. As indicated in previous studies, mammalian Ino80 is essential for development. Ex vivo studies showed that INO80 maintains embryonic stem cell self‐renewal by occupying regions proximal to the pluripotency gene promoters to activate their expression.^17^ Successful proximal‐distal axis establishment may be partly due to the inhibition of BMP4 expression by INO80.^18^ Deletion of mIno80 resulted in embryonic lethality.^16^ In addition, Ino80 KO inhibited cell proliferation and anchorage‐independent growth.^15^ Because INO80 is a well‐documented regulator of gene expression, embryonic developmental defects and adverse pregnancy outcomes may be caused by abnormal epigenetic regulation of INO80. However, the specific role of INO80 in blastocyst implantation and placenta formation has not yet been revealed.

Recently, INO80 was found to be involved in tumorigenesis by occupying superenhancers and promoting oncogenic transcription through facilitating nucleosome depletion and Mediator recruitment.^15^ In response to trophoblast cell biology and tumour cell similarity,^19^ we investigated whether INO80 is involved in the regulation of trophoblastic function. Here, we show that INO80 indeed plays an essential role in the occurrence of RM by regulating the transcription of invasion‐associated genes. Our data identify INO80‐mediated chromatin remodelling as a key player in trophoblast invasion and migration.

## 2 MATERIALS AND METHODS

### 2.1 Patient characteristics

Fifteen Patients with RM between 23 and 35 years of age, who had been treated at the Department of Obstetrics and Gynecology of the Renmin Hospital of Wuhan University, Wuhan, Hubei Province, PR China between March 2018 and September 2019, were included in this study. Gestational age was from 5 to 10 weeks (mean gestational age (8.1 ± 1.6)). B‐type ultrasonic inspection suggested intrauterine pregnancy. Written consent was obtained from the patients. Human protocols were approved by the Ethics Committee of Wuhan University (2018017).

An additional fifteen women between 23 and 35 years of age with normal early pregnancies were recruited as healthy controls. Gestational age was from 5 to 10 weeks (mean gestational age (7.9 ± 2.1)). B‐type ultrasonic inspection suggested intrauterine pregnancy and so did an early embryonic heartbeat. All these women had experienced previous pregnancies without any history of spontaneous abortion.

Patients with the following features were excluded: (a) uterine abnormalities or cervical insufficiency; (b) comprehensive hormonal status assessment to rule out luteal phase defects, hyperprolactinemia and hyperandrogenemia; (c) endocrine or metabolic diseases (diabetes, hyperthyroidism, and hypothyroidism); and (d) abnormal karyotype analysis of parents or previous abortion.

There were no significant differences in age, gestational age, chromosomal abnormalities or systemic or infectious diseases between the two groups.

### 2.2 Cell culture

The HTR8/SVneo cell line, which was derived from a first‐trimester human extravillous cytotrophoblast, was maintained in DMEM/F12 plus 10% foetal bovine serum with P/S antibiotics at 37°C in a 5% CO_2_ atmosphere. The growth medium was changed every 24 hours. Cells in the logarithmic growth phase were used for experiments.

### 2.3 RNA isolation, reverse transcription and real‐time qPCR

Total RNA was isolated from cells using the TRIzol reagent (Life Technologies, Grand Island, NY, USA), and 0.5 μg of total RNA was reverse transcribed to generate cDNA using the iScript cDNA Synthesis Kit (1708891, Bio‐Rad) according to the manufacturer's instructions. qPCRs were performed using the iTaq Universal SYBR Green supermix (1725121, Bio‐Rad) on the ABI QuantStudio 6 Flex Real‐Time PCR System. ACTB expression was used for normalization. Primers used in the study are listed in Table 1.

**TABLE 1:** Primers for real‐time qPCR

Gene	Primer	Sequences of primers (5′‐3′)
β‐Actin	F	CCA ACC GCG AGA AGA TGA
	R	CCA GAG GCG TAC AGG GAT AG
Ino80	F	GGC ATA ATG AAA TGC AGC AG
	R	TAA CCG GGA CCC CAA TTC
MMP2	F	CAAGTGGGACAAGAACCAGA
	R	CCAAAGTTGATCATGATGTC
TIMP1	F	CTTCTGCAATTCCGACCTCGT
	R	ACGCTGGTATAAGGTGGTCTG
TIMP2	F	GCTGCGAGTGCAAGATCAC
	R	TGGTGCCCGTTGATGTTCTTC
LAMC2	F	ATCTGATGGACCAGCCTCTCA
	R	AGCCTGGGTATTGTAGCAGC
PLAU	F	GATACGAACAGGCGAACTGTG
	R	TGCTGCCCTCCGAATTTCTT
SPARC	F	ATTGACGGGTACCTCTCCCA
	R	GAAAAAGCGGGTGGTGCAAT
L1CAM	F	AGATCCCCGAGGAATTGATGG
	R	TTATTGCTGGCAAAGCAGCG
CXCR4	F	AGGGGATCAGTATATACACTTCAGA
	R	AGGTGCAGCCTGTACTTGTC
S100A4	F	GGGCAAAGAGGGTGACAAGT
	R	GTCCTTTTCCCCAAGAAGCTG

### 2.4 Western blot analysis

Total protein was extracted from the cells using RIPA lysis buffer. Equal amounts of protein were loaded into each lane, resolved via SDS‐PAGE and electrophoretically transferred to PVDF membranes (Bio‐Rad), which were subsequently incubated for 1 hour at room temperature in 5% fat‐free milk dissolved in Tris‐buffered saline (TBS) containing 0.1% Tween‐20. That step was followed by incubation with anti‐INO80 (1:500, 18810‐1‐AP, Proteintech) or anti‐ACTB antibodies (1:2000, sc‐47778, Santa Cruz Biotechnology) overnight at 4°C. Then, the membranes were incubated with peroxidase‐conjugated goat anti‐rabbit or anti‐mouse secondary antibodies for 1 hour after being washed. Immunoreactive bands were visualized with a SuperSignal^®^ West Femto Trial Kit (Thermo Scientific, USA) detection system.

### 2.5 Immunofluorescence analysis

Villous tissue sections were deparaffinized and rehydrated. Sections were blocked with goat serum for 1 hour at room temperature, and incubated with anti‐INO80 antibody (1:200, Proteintech) at 4°C for 16 hours. After the sections were washed with PBS, they were incubated with secondary fluorescent antibodies for 1 hour (1:300) at 37°C in the dark (Alexa Fluor 594‐conjugated goat anti‐Rabbit IgG, Life Technology). Sections were finally counterstained with DAPI and then analysed using a fluorescence microscope (Leica DMi8 microscope, Leica Microsystems). Representative images from 3 HC samples and 3 RM samples are shown. Five fields of view were randomly selected for each tissue sample (Scale bar = 25 μm), and the average value was taken after counting statistics based on the fluorescent staining results.

### 2.6 Growth curves

After 48 hours of transfection, the cells were seeded into 6‐well plates at a density of 1 × 10^4^ cells / 2 mL per well and incubated at 37°C in 5% CO_2_. The cells were harvested and counted every 24 hours. Cell proliferation was assessed by cell growth curves.

### 2.7 Plate clone formation assay

Briefly, after 48 hours of transfection, cells were digested and 400 cells from each group were inoculated into a 100 mm culture dish. The culture medium was changed every 2 days. After the cells had incubated for 14 days at 37°C in 5% CO_2_, their colonies were visible to the naked eye. The surviving clones were counted after fixation with paraformaldehyde and staining with crystal violet. Clonal formation efficiency (%) = (number of clones / number of inoculated cells) × 100%.

### 2.8 Scratch‐wound healing and transwell assay

The HTR8/SVneo cell migration ability was assessed by scratch‐wound and transwell assays as described previously. Briefly, HTR8/SVneo cells were transduced with either the shNT or the shINO80 lentivirus. Three days after infection, cells were either subjected to a scratch with a pipette tip or were preplated in transwell chambers, and images were taken at indicated time‐points. The distance of the wound edge or cell number across the transwell chambers was measured by ZEN analysis software at 12 hours.

The Matrigel cell invasion assay was carried out as previously described. We used Invasion Chambers (354480, Corning), which were pre‐coated with extracellular matrix proteins. The next steps were as described above. The cell number of cells that moved across the transwell chambers was calculated by ZEN analysis software at 24 hours.

### 2.9 Chromatin immunoprecipitation followed by high‐throughput sequencing (ChIP‐seq) and ChIP‐qPCR

ChIP for INO80 was performed as described previously.^20^ Briefly, the HTR8/SVneo cells were fixed using 1% formaldehyde for 10 minutes, and 0.125 mol/L glycine was added to stop fixation. Cells were harvested, and DNA was fragmented to 300‐500 bp by sonication with a Covaris S220 sonicator. Immunoprecipitation was performed with antibodies conjugated to Dynabeads Protein G beads (1004D, Life Technologies). ChIP DNA was eluted, reverse cross‐linked, extracted with phenol/chloroform and precipitated.

For ChIP‐Seq, 1 ng ChIP DNA or input DNA was used to generate sequencing libraries using the Nextera XT DNA sample preparation Kit (FC‐131‐1024, Illumina). Libraries were sequenced on the NextSeq500 sequencer (Illumina) using the 35 nt paired‐end sequencing protocol. Two biological replicates were performed for each ChIP‐Seq sample, and reads were combined for analysis.

The resulting DNA was used for qPCR analysis. All the primer sequences used for ChIP‐qPCR are listed in Tables 2 and 3.

**TABLE 2:** Positive Primers for ChIP‐qPCR

Gene	Primer	Sequences of primers (5′‐3′)
MMP2	F	GCTACGATGGAGGCGCTAA
	R	CTAAACGTGGTTCCTTGAGGC
LAMC2	F	GGGGAATCTCGCACATTCCAGGCAAAG
	R	ACAGAGCACAAACCGGGCTGGAAAATC
PLAU	F	GAGTGCGCTCTTGCTTTGAC
	R	GTGGATGGAATCCGGAGGAC
SPARC	F	CTGCTGCCTAAACCGACTCA
	R	TGGGGGCCATTGGGTAATTC
L1CAM	F	ATTGGCCCCATTCGACCTTT
	R	TCCATTTTGCTTCCTCGCCT
CXCR4	F	GACCTCCCAGAGGCATTTCC
	R	CAGAGAGACGCGTTCCTAGC
S100A4	F	GAGAGCGGATACTGCCTTCC
	R	CTGCTAGTAACCGCTAGGGC

**TABLE 3:** Negative Primers for ChIP‐qPCR

Gene	Primer	Sequences of primers (5′‐3′)
MMP2	F	CTTCTGCAGCCATTGCCATC
	R	GGGCAGCTTTGACTGTACC
LAMC2	F	CCACTCTTCTCCTACCCCCA
	R	CTGAGCAGCTGAACCCGTTA
PLAU	F	CCAAAATGCTGTGTGCTGCT
	R	CCCTTAAGCTGGCTGGAACA
SPARC	F	CCCCTGTTGGTCTGGGTAAG
	R	GAGTGAGATGCTTGGCTGGT
L1CAM	F	GTTAAAGGGCAGAGGGGGAC
	R	ACTTAGGAATGCCCAAGCCC
CXCR4	F	GAAGAAAGCTAGGGCCTCGG
	R	GCCTGTTGGCTGCCTTACTA
S100A4	F	GAGGACCCTCAAGAGGAGGT
	R	CTTCAAGGCCATGTCTGGGT

### 2.10 RNA‐Seq

HTR8/SVneo cells were transduced with a lentivirus‐based non‐targeting (shNT) or INO80 shRNA (shINO80‐3). Total RNA was extracted from cells using the GeneJET RNA Purification Kit (K0732, Thermo Scientific) 3 days after infection. 1 μg of RNA was used to generate a sequencing library using the TruSeq RNA Library Prep Kit V2 (RS‐122‐2002, Illumina) according to the manufacturer's instructions. All libraries were sequenced on the NextSeq500 sequencer (FC‐404‐2005, Illumina) using the 35 nt paired‐end sequencing protocol.

### 2.11 Statistical analysis

All statistical analyses were performed using GraphPad Prism 5.0 (GraphPad Software, San Diego, USA). All results are expressed as the means ± SEM. Statistical analysis involved the use of Student's *t* test or one‐way ANOVA, as indicated in the manuscript. *P* < 0.05 was considered statistically significant.

## 3 RESULTS

### 3.1 Ino80 was determined to be an essential gene required for early mouse embryonic development

**FIGURE 1:**
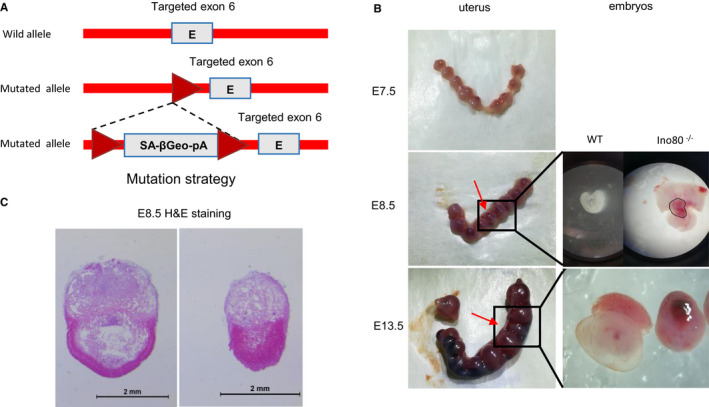
Ino80 was an essential gene required for early mouse embryonic development. (A) The mouse mutation strategy used in our study. (B) Representative images of uterus isolated from ± Ino80 mouse intercross breeding. Left image: numbers refer to individually implanted embryos. Embryos indicated by the red arrow were smaller and the genotyping revealed that they were Ino80−/−. Right images: isolated Ino80+/+ and Ino80−/− embryos at E8.5 and E10.5 showing defective morphology in Ino80−/− embryos. KO knockout, E embryonic day. (C) Representative images to show haematoxylin and eosin (HE) staining of Ino80+/+ and Ino80−/− embryos at E8.5. Scale bar = 2 mm

To investigate the in vivo effects of Ino80 in embryo development, we disrupted Ino80 in C57BL/6 mice by inserting a LACZ gene sequence with a polyA signal between the fifth and sixth protein‐coding exons using gene targeting (Figure 1A). Subsequently, we intercrossed heterozygous mice for the production of mouse embryos of different genotypes. To observe the differences between different embryonic phenotypes at various developmental stages, we continuously isolated embryos at E13.5 (mid‐gestation), E8.5 and E7.5. The results from these experiments showed that Ino80 KO homozygous embryos were absent at E13.5, indicating that the targeted disruption of Ino80 resulted in embryonic lethality. In general, there was no visible difference between the E7.5 embryos, whereas the Ino80 KO embryos were much smaller at E8.5 (Figure 1B). Embryo reabsorption likely occurs after E7.5 because Ino80 KO embryos at E8.5 begin to show a loss of structural integrity (Figure 1C). Thus, Ino80 is required for early mouse embryonic development.

### 3.2 Ino80 was found to be required for trophoblast viability, migration and invasion

**FIGURE 2:**
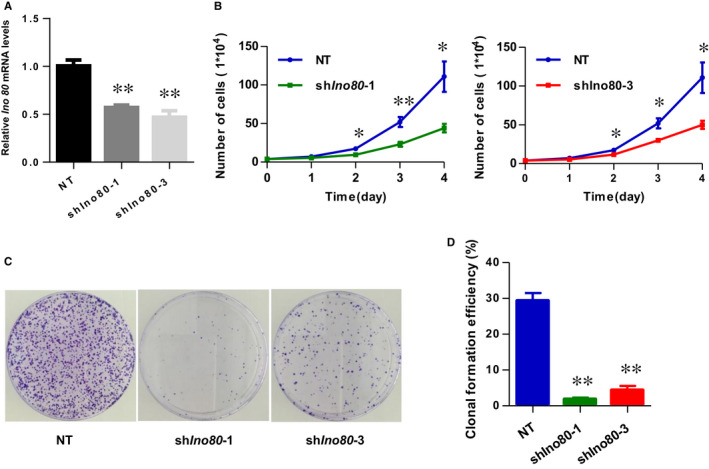
Ino80 was required for trophoblast cell viability. (A) Ino80 mRNA expression 3 d after Ino80 silencing. shIno80‐1 and shIno80‐3 represent two different shRNAs against Ino80. shNT represents non‐targeting shRNA control. Values were plotted as mean ± SEM from 3 independent experiments. *P* value was calculated by Student's *t* test. **P < 0.01. (B) Growth curve of HTR8/SVneo cells transfected with one of the above‐mentioned three vectors and incubated for 72 h. Counted every 24 h. Values were plotted as mean ± SEM from 3 independent experiments. *P* value was calculated by Student's *t* test. *P < 0.05, **P < 0.01. (C) Representative images to show plate clone formation assay of HTR8/SVneo cells transfected with one of the above‐mentioned three vectors and harvested after 14 d. (D) The number of clone formation was counted. Values were plotted as mean ± SEM from 3 independent experiments. *P* value was calculated by Student's *t* test. **P < 0.01

To define the role of the INO80 complex in survival capacity, migration and invasion of trophoblasts, we silenced Ino80 with lentivirus‐based shRNA in HTR8/SVneo cells. To reduce the possibility of potential off‐target effects, 2 individuals Ino80 shRNAs were used in our experiments. Both hairpins effectively suppressed the expression of Ino80 (Figure 2A).

**FIGURE 3:**
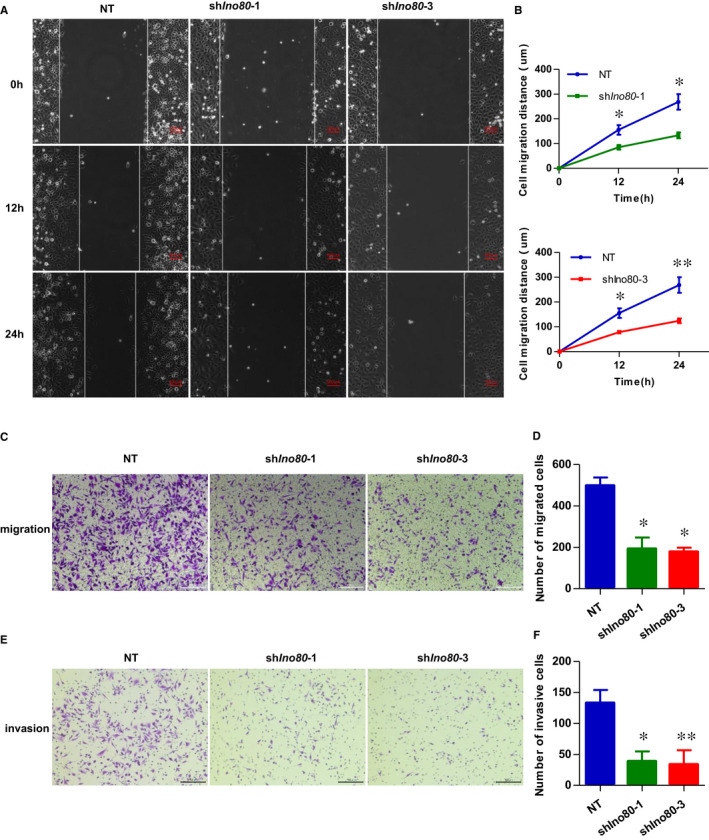
Ino80 was required for trophoblast migration and invasion. (A) Representative images to show scratch‐wound assay. Confluent HTR8/SVneo monolayers were transfected with either shIno80‐1, shIno80‐3 or shNT and subjected to scratch‐wound assay 3 d after infection. Images were taken 0, 12 and 24 h after assay (white lines indicate wound edge). Scale bar = 100 μm. (B) Quantitative analysis of migration distance. The mean distance was calculated from average of 6 microscope fields from 3 independent experiments. Values were plotted as mean ± SEM. *P* value was calculated by Student's *t* test. *P < 0.05, **P < 0.01. (C) Representative images to show HTR8/SVneo cells migration in transwell chambers. HTR8/SVneo cells were transfected with either shIno80‐1, shIno80‐3 or shNT, respectively. Scale bar = 200 μm. (D) Quantitative analysis of the number of migrated cells. Migrated cells were quantified by the average of 5 randomly selected regions per experiment, and from 3 independent experiments. Values were plotted as mean ± SEM. *P* value was calculated by Student's *t* test. *P < .05. (E) Representative images to show HTR8/SVneo cells invasion in transwell chambers, which were pre‐coated with extracellular matrix proteins. The HTR8/SVneo cells were transfected with either shIno80‐1, shIno80‐3 or shNT, respectively. Scale bar = 200 μm. (F) Quantitative analysis of the number of invasion cells. Invaded cells were quantified by the average of 5 randomly selected regions per experiment, and from 3 independent experiments. Values were plotted as mean ± SEM. *P* value was calculated by Student's *t* test. *P < 0.05, **P < 0.01

The Plate Cell Clone Formation and Growth Curve assay demonstrated that knockdown of INO80 decreased HTR8/SVneo proliferation activity and viability (Figure 2B,C). In vitro scratch‐wound healing assays revealed that knockdown of Ino80 markedly reduced trophoblast cell migration (Figure 3A,B). Transwell migration assays further confirmed a significant reduction of migration at 24 hours in Ino80‐depleted cells compared with control cells (Figure 3C,D). More importantly, the invasive ability of Ino80‐depleted cells was also significantly weakened. Together, these observations demonstrated a clear correlation between Ino80 expression and trophoblast cell viability, migration and invasion.

### 3.3 Ino80 regulated the expression of invasion‐associated genes

**FIGURE 4:**
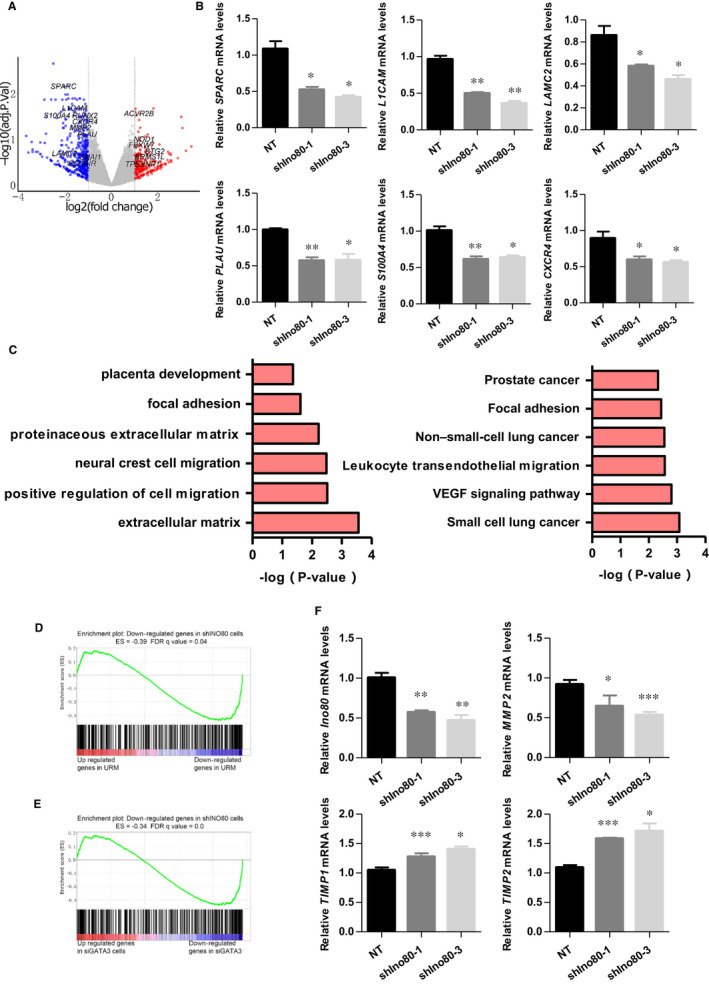
Ino80 regulated the expression of invasion‐related genes. (A) Volcano plot to show gene expression changes on Ino80 silencing in HTR8/SVneo cells. Cells were collected 3 d after Ino80 silencing and total RNA was prepared for RNA‐Seq. (B) Expressions of SPARC, L1CAM, LAMC2, PLAU, S100A4 and CXCR4 were determined by real‐time PCR in HTR8/SVneo cells transfected with either shIno80‐1, shIno80‐3 or shNT, respectively. Values were plotted as mean ± SEM from 3 independent experiments. *P* value was calculated by Student's *t* test. *P < 0.05, **P < 0.01. (C) Gene ontology analysis and KEGG analysis of genes down‐regulated on Ino80 depletion. Selected 6 categories are shown. (D) Gene set enrichment analysis (GSEA) showing that Ino80 silencing repressed genes were enriched for genes that were low expressed in villus tissues of URM. (E) GSEA showing that Ino80 silencing‐repressed genes were enriched for genes that were low expressed in trophoblasts after GATA3 knock‐down. (F) Expressions of Ino80, MMP2, TIMP1 and TIMP2 were determined by real‐time PCR in HTR8/SVneo cells transfected with either shIno80‐1, shIno80‐3 or shNT, respectively. Values were plotted as mean ± SEM from 3 independent experiments. *P* value was calculated by Student's *t* test. *P < 0.05, **P < 0.01, ***P < 0.001

To understand how INO80 regulates trophoblast migration and invasion at the molecular level, we carried out RNA high‐throughput sequencing (RNA‐seq) upon Ino80 silencing in HTR‐8/SVneo 3 days post‐infection. We found that nearly two‐thirds (560/863) of differentially expressed genes (DEGs) were down‐regulated upon Ino80 silencing, including many known to play important roles in cell invasion such as SPARC, L1CAM, LAMC2, PLAU, S100A4 and CXCR4 (Figure 4A,B). An unbiased survey of the functions of Ino80 silencing‐repressed genes by both gene ontology (GO) analysis and KEGG analysis identified significant enrichment in physiological processes related to cancer, extracellular matrix and focal adhesion, reinforcing the link between Ino80 and cell invasion (Figure 4C). Furthermore, gene set enrichment analysis (GSEA) showed that the Ino80 silencing‐repressed genes share a high degree of overlap with genes that are down‐regulated in RM tissues compared with normal controls (Figure 4D). A previous study showed that knocking down GATA3 in trophoblast cells resulted in reduced cell migration and invasion.^21^ Next, we directly compared global gene expression changes caused by Ino80 silencing and GATA3 KD. Ino80 silencing clustered most closely with siGATA3 treatment (Figure 4E, Supplementary Material, Figure S1).

Proteolysis of the extracellular matrix (ECM) plays a crucial role in the regulation of cell motility, which depends on the dynamic balance between MMPs and TIMPs. MMP2 has been implicated in remodelling the ECM during the process of trophoblast invasion.^22^ As shown in Figure 4F, MMP2 expression was decreased in the trophoblast cells transfected with shIno80, whereas the levels of TIMP1 and TIMP2, which are the major endogenous inhibitors of MMP activity, were noticeably increased.

Taken together, these results point to a pivotal role for Ino80 in regulating trophoblast migration and invasion, and the occurrence of miscarriage.

### 3.4 Ino80 occupied genomic regions near invasion‐associated genes

**FIGURE 5:**
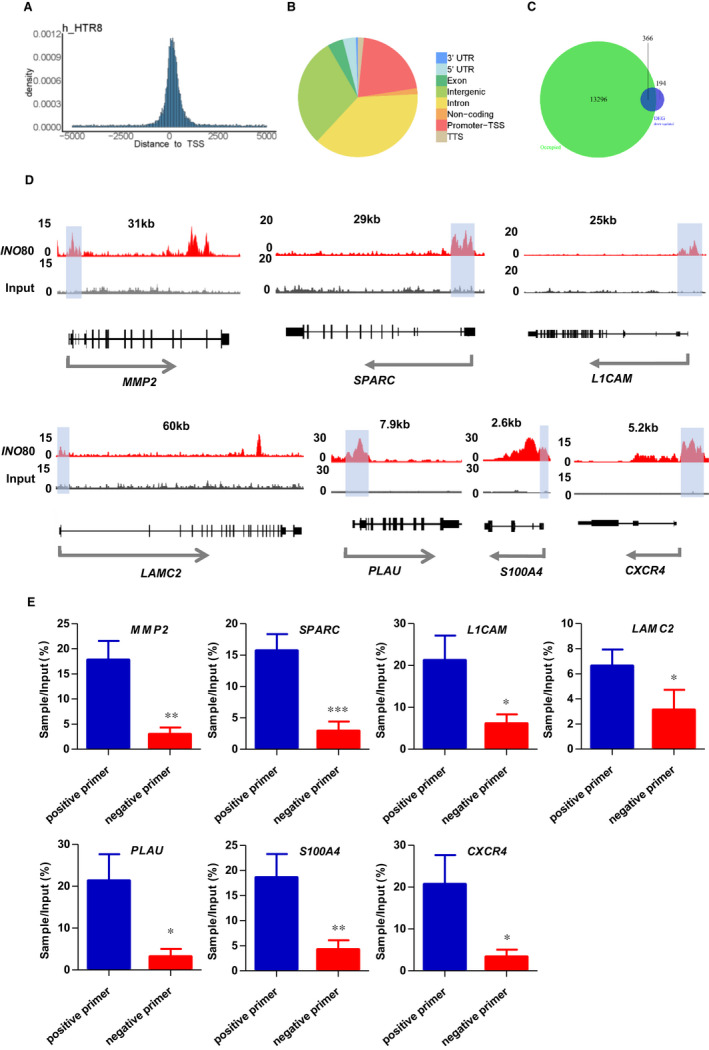
Ino80 occupied genomic regions near invasion‐associated genes. (A, B) Ino80 peak distribution in the genome. (C) Venn diagram to show the overlaps between Ino80 chromatin immunoprecipitation (ChIP) signal and Ino80 knock‐down‐repressed genes.(D) Genome tracks displaying Ino80 occupancy near selected invasion‐related genes, MMP2, et al, in HTR8/Svneo cells. (E) ChIP‐qPCR showing the enrichment of Ino80 on the chromatin of selected invasion‐related genes. One pair of positive primers and a pair of negative primers were designed near TSS to verify binding, respectively. ChIP‐qPCR data are the average of three biological replicates. Values were plotted as mean ± SEM. *P* value was calculated by Student's *t* test. *P < 0.05; **P < 0.01; ***P < 0.001

The fact that invasion‐associated genes such as MMP2, SPARC, L1CAM, LAMC2, PLAU, S100A4 and CXCR4 were quickly down‐regulated after Ino80 silencing in HTR8/SVneo cells (Figure 4B,F) suggested that the complex may directly regulate their expression. To test this hypothesis, we performed chromatin immunoprecipitation followed by high‐throughput sequencing (ChIP‐seq) to examine the genomic localization of Ino80 peaks and compared Ino80 localization with transcriptional regulatory sequences such as promoters and enhancers. We found that Ino80 peaks were enriched in gene‐rich chromosomal regions near transcription start sites (promoter‐TSS; Figure 5A,B, Supplementary Material, Figure S2A). Furthermore, of the 863 genes that showed differential expression after Ino80 silencing, 518 had nearby Ino80 occupancy and were likely its direct targets (Figure 5C, Supplementary Material, Figure S2B). A total of 366 genes were significantly down‐regulated due to the loss of Ino80 occupancy (Figure 5C). Inspection of individual gene tracks and ChIP followed by quantitative PCR (ChIP‐qPCR) showed that Ino80 occupied genomic regions near well‐characterized invasion‐associated genes (Figure 5D,E). Together, these results support the model that INO80 directly occupies promoter regions to affect the expression of invasion‐associated genes in trophoblast cells.

### 3.5 INO80 was decreased in chorionic villous tissues of recurrent miscarriage patients

**FIGURE 6:**
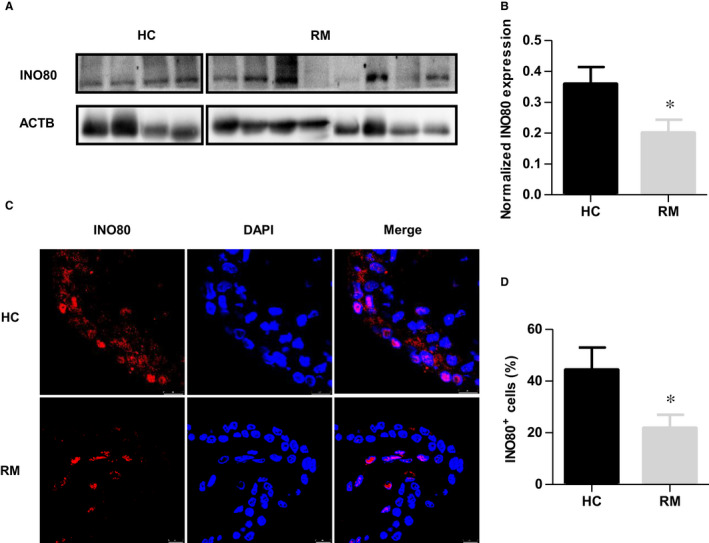
INO80 was decreased in chorionic villous tissues of recurrent miscarriage patients. (A) The INO80 protein levels in first‐trimester villous tissues from the HC and RM groups were determined by Western blotting. (B) Quantitative analysis of Western blots. Expression level was normalized by ACTB; n = 15. Values were plotted as mean ± SEM; *P* value was calculated by Student's *t* test. *P < 0.05. (C) Representative immunofluorescence images of INO80 in first‐trimester villous sections of RM and HC samples. Red indicates fluorescence signals specific to anti‐INO80 antibodies, and blue indicates nuclei. Scale bar = 25 μm. (D) The number of INO80 + cells was calculated, respectively. Then, the percentage of INO80 + cells normalized to the number of nuclei in the villous tissue of RM and HC samples was assessed; n = 15. Values were plotted as mean ± SEM; *P* value was calculated by Student's *t* test. *P < 0.05

First‐trimester human chorionic villous tissues were collected to investigate whether INO80 is involved in the pathogenesis of RM. Our Western blotting analyses showed that INO80 protein levels were significantly decreased in RM patients compared with healthy controls (Figure 6A,B). In addition, immunofluorescence staining was used to confirm the expression of INO80 in the paraffin‐embedded sections from first‐trimester human chorionic villous tissue. Fluorescence staining with an anti‐INO80 antibody revealed that the INO80 was reduced in RM patients compared with healthy controls (Figure 6C,D). Collectively, our results indicated a potential role for decreased expression of the INO80 chromatin remodelling complex in the occurrence of miscarriage.

## 4 DISCUSSION

The rate of RM gradually increases with the increasing maternal age.^4^ Furthermore, pregnancy complications tend to recur, so that an occurrence in a first pregnancy predisposes that same occurrence in a second pregnancy.^23^ RM is also associated with serious psychological distress and an emotional burden for couples. However, the mechanisms underlying RM remain poorly understood. In this study, we found that Ino80 KO embryos fail to survive and that the expression level of INO80 is significantly decreased in RM patient samples compared with normal samples, suggesting that INO80 may be involved in the pathogenesis of RM.

In recent years, numerous studies have established a strong link between epigenetic regulation and placental development in maintaining a healthy pregnancy. Uterine environmental disorders and placental dysplasia caused by abnormal epigenetic regulation led to severe pregnancy‐related diseases.^24^, ^25^ Although several genome‐wide types of epigenetic regulation (eg DNA methylation,^3^ histone modifications ^26^) participate in many aspects of early pregnancy, including decidualization,^27^ trophoblast differentiation and invasion, and placental imprinted gene expression,^28^ the role of chromatin remodelling complexes, such INO80, have not been clearly defined in these processes. In addition to the functions of DNA replication and repair, telomere maintenance and chromosome segregation, INO80 silencing reduces oncogenic transcription.^28^, ^29^ Trophoblast migration and invasion are often analogized to tumour metastasis, and they share many common molecular mechanisms. Therefore, we explored the relationship between INO80 and invasion‐associated genes in trophoblasts. Based on RNA‐seq and ChIP‐seq analysis, we found that Ino80 is enriched at the promoter region of well‐characterized invasion‐associated genes, such as MMP2, SPARC, L1CAM, LAMC2, PLAU, S100A4 and CXCR4. Knockdown of Ino80 in trophoblasts significantly reduced the expression of these genes. Therefore, we propose that INO80 regulates the invasive ability of trophoblasts by regulating a complex invasion‐associated gene network.

For implantation to occur and placental formation, trophoblast stem (TS) cells must undergo differentiation, with the acquisition of a mesenchymal morphology and invasive properties. The commitment of stem cells differentiate into to specialized cell types requires extensive reprogramming of gene expression, which partially involves changes to the epigenetic control of transcription.^26^, ^30^ Given the role of INO80 in trophoblast invasion, we suspect that it may also be involved in TS cell differentiation. However, specific molecular mechanisms require further exploration in future studies.

Invasion of trophoblasts into the endometrial stroma and inner‐third of the myometrium is essential for the development of placenta and pregnancy success in humans.^31^ Recent studies have revealed that accurate regulation of MMP activity at the maternal‐foetal interface appears to be critical for trophoblast invasion and migration in the first trimester, whereas disordered regulation may result in failed implantation, impaired placental perfusion and chronic placental ischaemia in gestation leading to RM.^32^ Trophoblasts constitutively produce MMPs and are therefore intrinsically invasive. In particular, MMP2 has been implicated in the remodelling of the ECM during the trophoblast invasion process.^33^ Here, we first found that in human trophoblasts, Ino80 was recruited and bound to the promoter region of MMP2 and further promoted the expression of MMP2. TIMPs, which are the major endogenous inhibitors of MMP activity in tissues, inhibit trophoblast invasion. In this study, knock‐down of Ino80 increased the expression of TIMP1 and TIMP2, which in turn inhibited MMP2 activity and decreased trophoblast invasion. These findings further highlight the role of INO80 in trophoblast invasion and the RM process.

A comprehensive delineation of epigenetic mechanisms involved in placentation will allow for a better understanding of the complex molecular events associated with human implantation and placental development. The regulation of chromatin remodelling complexes is a new natural perspective yet to be explored. In the present study, we demonstrated that INO80 is vital for embryo survival. Interestingly, notably higher levels of INO80 were produced by trophoblasts in normal samples compared with the RM samples. In addition, knock‐down of Ino80 effectively inhibited trophoblast migration and invasion in the HTR‐8/SVneo cell model, which was accompanied by less MMP2 and more TIMP production by trophoblasts compared with controls. As mentioned earlier, Ino80 bound to genomic regions near invasion‐associated genes, suggesting that INO80 may directly regulate the expression of key genes essential for trophoblastic invasion. The correlation between the decreased expression of INO80 and RM may serve as a diagnostic marker and therapeutic target in RM patients. A deeper understanding of the epigenetic regulatory network of early stages of gestation will open a new avenue to improve or abrogate this critical reproductive event and lead to healthy foetal outcomes.

## CONFLICT OF INTEREST

The authors confirm that there are no conflicts of interest.

## AUTHOR CONTRIBUTIONS

**Shu Xian:** Conceptualization (lead); Data curation (equal); Methodology (equal). **Yan Zhang:** Data curation (equal); Methodology (equal). **Li Wang:** Investigation (equal). **Fang Yao:** Formal analysis (equal). **Jinli Ding:** Formal analysis (equal). **yanqing wang:** Methodology (equal). **Xiao Yang:** Formal analysis (equal). **fangfang dai:** Investigation (equal). **Tailang Yin:** Conceptualization (equal); Project administration (equal). **Yanxiang Cheng:** Funding acquisition (equal); Project administration (equal).

## Supporting information

Fig S1

Fig S2

## Data Availability

The data sets and supporting materials generated and/ or analysed during the current study are available from the corresponding author on reasonable request.

## References

[1] Norwitz ER , Schust DJ , Fisher SJ . Implantation and the survival of early pregnancy. N Engl J Med. 2001;345(19):1400‐1408. 11794174 10.1056/NEJMra000763

[2] Dwi Putra SE , Reichetzeder C , Meixner M , Liere K , Slowinski T , Hocher B . DNA methylation of the glucocorticoid receptor gene promoter in the placenta is associated with blood pressure regulation in human pregnancy. J Hypertens. 2017;35(11):2276‐2286. 28817493 10.1097/HJH.0000000000001450

[3] Yu M , Du G , Xu Q , et al. Integrated analysis of DNA methylome and transcriptome identified CREB5 as a novel risk gene contributing to recurrent pregnancy loss. EBioMedicine. 2018;35:334‐344. 30100398 10.1016/j.ebiom.2018.07.042 PMC6154871

[4] Practice Committee of the American Society for Reproductive M . Evaluation and treatment of recurrent pregnancy loss: a committee opinion. Fertil Steril. 2012;98(5):1103‐1111. 22835448 10.1016/j.fertnstert.2012.06.048

[5] El Hachem H , Crepaux V , May‐Panloup P , Descamps P , Legendre G , Bouet PE . Recurrent pregnancy loss: current perspectives. Int J Womens Health. 2017;9:331‐345. 28553146 10.2147/IJWH.S100817 PMC5440030

[6] Branch DW , Gibson M , Silver RM . Clinical practice. Recurrent miscarriage. N Engl J Med. 2010;363(18):1740‐1747. 20979474 10.1056/NEJMcp1005330

[7] Diejomaoh MF . Recurrent spontaneous miscarriage is still a challenging diagnostic and therapeutic quagmire. Med Princ Pract. 2015;24(Suppl 1):38‐55. 25428171 10.1159/000365973 PMC6489083

[8] Liu Y , Fan X , Wang R , et al. Single‐cell RNA‐seq reveals the diversity of trophoblast subtypes and patterns of differentiation in the human placenta. Cell Res. 2018;28(8):819‐832. 30042384 10.1038/s41422-018-0066-y PMC6082907

[9] Pollheimer J , Vondra S , Baltayeva J , Beristain AG , Knofler M . Regulation of placental extravillous trophoblasts by the maternal uterine environment. Front Immunol. 2018;9:2597. 30483261 10.3389/fimmu.2018.02597 PMC6243063

[10] Zhao HJ , Klausen C , Li Y , Zhu H , Wang YL , Leung PCK . Bone morphogenetic protein 2 promotes human trophoblast cell invasion by upregulating N‐cadherin via non‐canonical SMAD2/3 signaling. Cell Death Dis. 2018;9(2):174. 29416020 10.1038/s41419-017-0230-1 PMC5833391

[11] Becker PB , Workman JL . Nucleosome remodeling and epigenetics. Cold Spring Harb Perspect Biol. 2013;5(9):a017905. 24003213 10.1101/cshperspect.a017905 PMC3753709

[12] Gerhold CB , Gasser SM . INO80 and SWR complexes: relating structure to function in chromatin remodeling. Trends Cell Biol. 2014;24(11):619‐631. 25088669 10.1016/j.tcb.2014.06.004

[13] Bao Y , Shen X . Chromatin remodeling in DNA double‐strand break repair. Curr Opin Genet Dev. 2007;17(2):126‐131. 17320375 10.1016/j.gde.2007.02.010

[14] Hur SK , Park EJ , Han JE , et al. Roles of human INO80 chromatin remodeling enzyme in DNA replication and chromosome segregation suppress genome instability. Cell Mol Life Sci. 2010;67(13):2283‐2296. 20237820 10.1007/s00018-010-0337-3 PMC11115786

[15] Zhou B , Wang L , Zhang S , et al. INO80 governs superenhancer‐mediated oncogenic transcription and tumor growth in melanoma. Genes Dev. 2016;30(12):1440‐1453. 27340176 10.1101/gad.277178.115 PMC4926866

[16] Min JN , Tian Y , Xiao Y , Wu L , Li L , Chang S . The mINO80 chromatin remodeling complex is required for efficient telomere replication and maintenance of genome stability. Cell Res. 2013;23(12):1396‐1413. 23979016 10.1038/cr.2013.113 PMC3847565

[17] Wang L , Du Y , Ward JM , et al. INO80 facilitates pluripotency gene activation in embryonic stem cell self‐renewal, reprogramming, and blastocyst development. Cell Stem Cell. 2014;14(5):575‐591. 24792115 10.1016/j.stem.2014.02.013 PMC4154226

[18] Qiu Z , Elsayed Z , Peterkin V , Alkatib S , Bennett D , Landry JW . Ino80 is essential for proximal‐distal axis asymmetry in part by regulating Bmp4 expression. BMC Biol. 2016;14:18. 26975355 10.1186/s12915-016-0238-5 PMC4790052

[19] Holtan SG , Creedon DJ , Haluska P , Markovic SN . Cancer and pregnancy: parallels in growth, invasion, and immune modulation and implications for cancer therapeutic agents. Mayo Clin Proc. 2009;84(11):985‐1000. 19880689 10.1016/S0025-6196(11)60669-1 PMC2770910

[20] Whyte WA , Orlando DA , Hnisz D , et al. Master transcription factors and mediator establish super‐enhancers at key cell identity genes. Cell. 2013;153(2):307‐319. 23582322 10.1016/j.cell.2013.03.035 PMC3653129

[21] Lee B , Kroener LL , Xu N , et al. Function and hormonal regulation of GATA3 in human first trimester placentation. Biol Reprod. 2016;95(5):113. 27733378 10.1095/biolreprod.116.141861 PMC5178150

[22] Zhang Y , Jin F , Li XC , et al. The YY1‐HOTAIR‐MMP2 signaling axis controls trophoblast invasion at the maternal‐fetal interface. Mol Ther. 2017;25(10):2394‐2403. 28750739 10.1016/j.ymthe.2017.06.028 PMC5628865

[23] Lykke JA , Paidas MJ , Langhoff‐Roos J . Recurring complications in second pregnancy. Obstet Gynecol. 2009;113(6):1217‐1224. 19461415 10.1097/AOG.0b013e3181a66f2d

[24] Li X , Wu C , Shen Y , et al. Ten‐eleven translocation 2 demethylates the MMP9 promoter, and its down‐regulation in preeclampsia impairs trophoblast migration and invasion. J Biol Chem. 2018;293(26):10059‐10070. 29773648 10.1074/jbc.RA117.001265 PMC6028950

[25] Huang Z , Du G , Huang X , et al. The enhancer RNA lnc‐SLC4A1‐1 epigenetically regulates unexplained recurrent pregnancy loss (URPL) by activating CXCL8 and NF‐kB pathway. EBioMedicine. 2018;38:162‐170. 30448228 10.1016/j.ebiom.2018.11.015 PMC6306333

[26] Abell AN , Jordan NV , Huang W , et al. MAP3K4/CBP‐regulated H2B acetylation controls epithelial‐mesenchymal transition in trophoblast stem cells. Cell Stem Cell. 2011;8(5):525‐537. 21549327 10.1016/j.stem.2011.03.008 PMC3091002

[27] Maekawa R , Tamura I , Shinagawa M , et al. Genome‐wide DNA methylation analysis revealed stable DNA methylation status during decidualization in human endometrial stromal cells. BMC Genom. 2019;20(1):324. 10.1186/s12864-019-5695-0 PMC6489213 31035926

[28] Zadora J , Singh M , Herse F , et al. Disturbed placental imprinting in preeclampsia leads to altered expression of DLX5, a human‐specific early trophoblast marker. Circulation. 2017;136(19):1824‐1839. 28904069 10.1161/CIRCULATIONAHA.117.028110 PMC5671803

[29] Zhang S , Zhou B , Wang L , et al. INO80 is required for oncogenic transcription and tumor growth in non‐small cell lung cancer. Oncogene. 2017;36(10):1430‐1439. 27641337 10.1038/onc.2016.311 PMC6534818

[30] Tanaka S , Kunath T , Hadjantonakis AK , Nagy A , Rossant J . Promotion of trophoblast stem cell proliferation by FGF4. Science. 1998;282(5396):2072‐2075. 9851926 10.1126/science.282.5396.2072

[31] Staun‐Ram E , Shalev E . Human trophoblast function during the implantation process. Reprod Biol Endocrinol. 2005;3:56. 16236179 10.1186/1477-7827-3-56 PMC1289292

[32] Tian FJ , Cheng YX , Li XC , et al. The YY1/MMP2 axis promotes trophoblast invasion at the maternal‐fetal interface. J Pathol. 2016;239(1):36‐47. 27071480 10.1002/path.4694 PMC5071713

[33] Zhu JY , Pang ZJ , Yu YH . Regulation of trophoblast invasion: the role of matrix metalloproteinases. Rev Obstet Gynecol. 2012;5(3‐4):e137‐e143. 23483768 PMC3594863

