# Supplementary material for: INO80 participates in the pathogenesis of recurrent miscarriage by epigenetically regulating trophoblast migration and invasion
Source: J Cell Mol Med. 2021 Mar 16;25(8):3885–97. doi: 10.1111/jcmm.16322 (PMC8051727; doi:10.1111/jcmm.16322)
Supplement: Supplementary file 1 — Fig S1 [file JCMM-25-3885-s002.pdf]

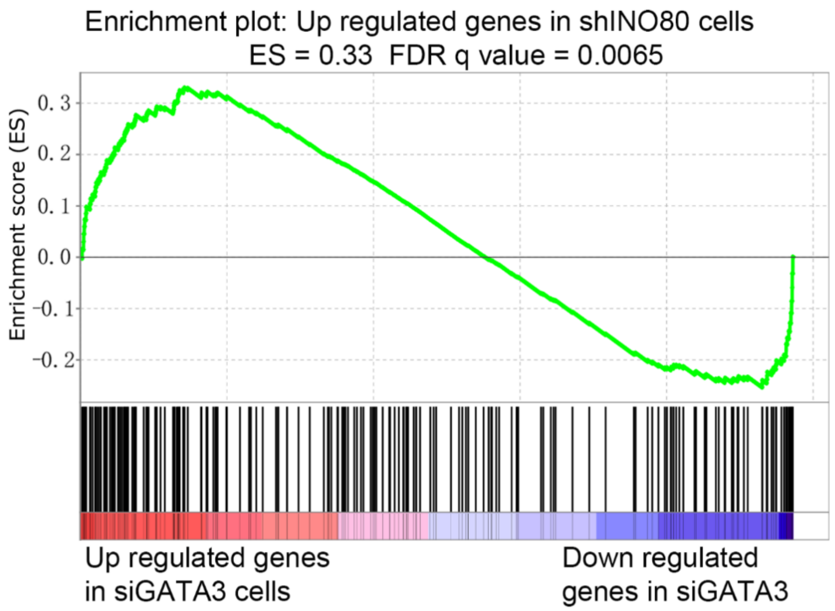

FIGURE S1. Gene set enrichment analysis (GSEA) showing that INO80 silencing-upregulated genes were enriched for genes that were high expressed in villus tissues of URM.
