# Supplementary material for: INO80 participates in the pathogenesis of recurrent miscarriage by epigenetically regulating trophoblast migration and invasion
Source: J Cell Mol Med. 2021 Mar 16;25(8):3885–97. doi: 10.1111/jcmm.16322 (PMC8051727; doi:10.1111/jcmm.16322)
Supplement: Supplementary file 2 — Fig S2 [file JCMM-25-3885-s001.pdf]

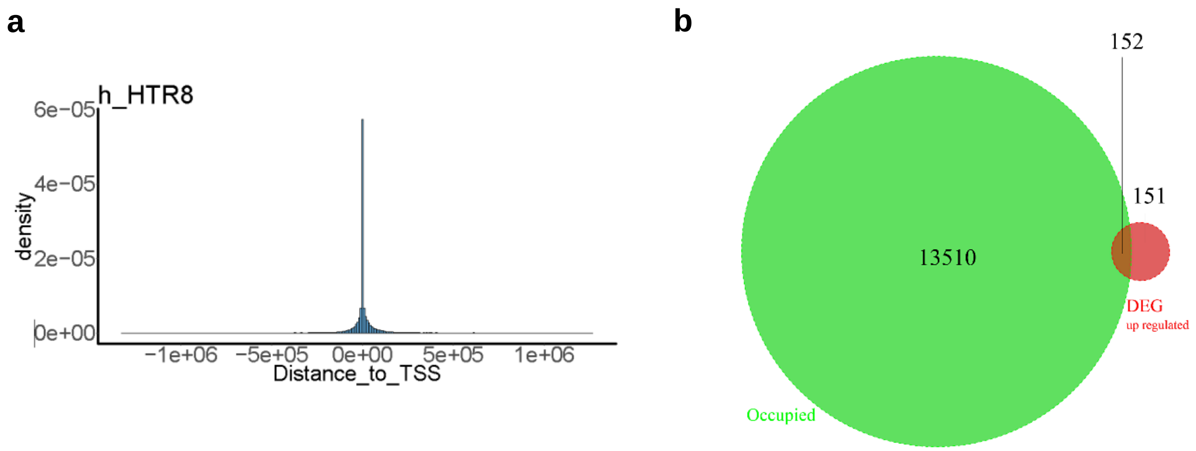

FIGURE S2. (a) Ino80 peak distribution in the genome. (b) Venn diagram to show the overlaps between INO80 chromatin immunoprecipitation (ChIP) signal and INO80 knockdown-upregulated genes.
